# Modified *Fusarium* Mycotoxins in Cereals and Their Products—Metabolism, Occurrence, and Toxicity: An Updated Review

**DOI:** 10.3390/molecules23040963

**Published:** 2018-04-20

**Authors:** Marcin Bryła, Agnieszka Waśkiewicz, Edyta Ksieniewicz-Woźniak, Krystyna Szymczyk, Renata Jędrzejczak

**Affiliations:** 1 Department of Food Analysis, Prof. Waclaw Dabrowski Institute of Agricultural and Food Biotechnology, Rakowiecka 36, 02-532 Warsaw, Poland; edyta.wozniak@ibprs.pl (E.K.-W.); krystyna.szymczyk@ibprs.pl (K.S.); renata.jedrzejczak@ibprs.pl (R.J.); 2 Department of Chemistry, Poznan University of Life Sciences, Wojska Polskiego 75, 60-625 Poznan, Poland; agnieszka.waskiewicz@up.poznan.pl

**Keywords:** modified mycotoxins, toxicity, occurrence, cereals

## Abstract

Mycotoxins are secondary fungal metabolites, toxic to humans, animals and plants. Under the influence of various factors, mycotoxins may undergo modifications of their chemical structure. One of the methods of mycotoxin modification is a transformation occurring in plant cells or under the influence of fungal enzymes. This paper reviews the current knowledge on the natural occurrence of the most important trichothecenes and zearalenone in cereals/cereal products, their metabolism, and the potential toxicity of the metabolites. Only very limited data are available for the majority of the identified mycotoxins. Most studies concern biologically modified trichothecenes, mainly deoxynivalenol-3-glucoside, which is less toxic than its parent compound (deoxynivalenol). It is resistant to the digestion processes within the gastrointestinal tract and is not absorbed by the intestinal epithelium; however, it may be hydrolysed to free deoxynivalenol or deepoxy-deoxynivalenol by the intestinal microflora. Only one zearalenone derivative, zearalenone-14-glucoside, has been extensively studied. It appears to be more reactive than deoxynivalenol-3-glucoside. It may be readily hydrolysed to free zearalenone, and the carbonyl group in its molecule may be easily reduced to α/β-zearalenol and/or other unspecified metabolites. Other derivatives of deoxynivalenol and zearalenone are poorly characterised. Moreover, other derivatives such as glycosides of T-2 and HT-2 toxins have only recently been investigated; thus, the data related to their toxicological profile and occurrence are sporadic. The topics described in this study are crucial to ensure food and feed safety, which will be assisted by the provision of widespread access to such studies and obtained results.

## 1. Introduction

Mycotoxins are natural, secondary metabolites produced by various fungi species. These compounds are significantly different from most synthetic food contaminants (e.g., residues of veterinary drugs, pesticides, and environmental contamination); their presence in food is almost unavoidable and depends strongly on climatic conditions, control of which is difficult or even impossible. Thus, the hazards to human and animal health posed by these natural substances may be more significant than the hazards produced by humans as a result of pesticide use in plant protection, veterinary drugs used in protection of animal health, preservatives, or food additives [1]. The only ways to maintain the lowest possible levels of these substances in food are to observe good agricultural practices, ensure variation of crops, use plant varieties resistant to fungal diseases, and apply fungicides. The restrictions on synthetic fungicides have led to the increased use of natural fungicides, such as metabolites from plants, including phenolic compounds and essential oils [2,3,4].

The most important fungi-producing metabolites that are critical for the maintenance of food safety include *Aspergillus*, *Fusarium*, and *Penicillium*. Fungi, as phytopathogens, infect plants during their growth and colonise plants after their harvest [5]. At favourable temperature and humidity, the fungi synthesise mycotoxins. The most important mycotoxins whose content in food is legally regulated include aflatoxins, ochratoxin A, zearalenone (ZEN), deoxynivalenol (DON), and fumonisins. The first group of toxins is produced by *Aspergillus*, the second by *Aspergillus* and *Penicillium*, and the others by *Fusarium*. 

*Fusarium* fungi are mainly responsible for contamination of cereals and the related risks to human and animal health [6,7]. Moreover, seed grain infected with fungi is of poorer quality and has lower yield, so the economic consequences may be significant: it is estimated that approximately 25% of global cereal production and approximately 20% of global plant production may be contaminated with mycotoxins, although the contamination level may vary significantly depending on local weather conditions [8,9]. In the EU permissible levels of mycotoxins were regulated by the European Commission on 19 December 2006 (1881/2006 Regulation with later amendments). *Fusarium* mycotoxin limits specified in unprocessed cereals, milling products, and cereal foodstuffs are (depending on the matrix): 200–1750 µg/kg for DON, 20–400 µg/kg for ZEN, 200–4000 µg/kg for the sum of B1 + B2 fumonisins (FB1 + FB2 combined) [10]. Furthermore, 15–1000 µg/kg limits have been recommended for the sum of HT2 and T-2 toxins in various matrices [11].

The complexity of the food safety problem increases if so-called “modified mycotoxins” are taken into account. Many aspects, including the occurrence/co-occurrence of these compounds with their basic analogues and their toxicological properties, are unknown. The “modified mycotoxins” term was introduced by Rychlik et al. [12] to refer to any mycotoxin whose structure has been changed in the course of some chemical/biochemical reaction [13]. Possible ways to modify mycotoxins are numerous. In particular, biologically modified mycotoxins are biosynthesised under the influence of some plant/animal/bacterial enzymes. Many of these substances have been extensively studied in terms of their formation path, toxicological properties, and occurrence in food. “Masked mycotoxins” is another term often used; it refers to a narrower group of mycotoxins produced in the course of some detoxication reactions run by plant organisms in an attempt to neutralise the toxins (II phase metabolites) [12]. The term was introduced by Gareis et al. [14], who demonstrated the decomposition of zearalenone-14-glucoside (ZEN-14Glc) during digestion of feed in the porcine gastrointestinal tract. Under favourable conditions (e.g., an acidic environment inside the stomach or the right enzymes in the small intestine and intestinal bacteria), similar decomposition may also take place in the human gastrointestinal tract. It has been recommended that the “masked mycotoxins” term be used only to refer to plant metabolites of mycotoxins formed in some phase II reactions, excluding otherwise modified mycotoxins [15].

Amongst the many possible mycotoxin modifications, biologically modified mycotoxins are a special group of compounds that are biosynthesised under the influence of plant, animal, or bacterial enzymes. Many of these substances are currently studied in terms of the path of their formation, their toxicological properties, and their presence in food. This work presents the current knowledge on the occurrence of certain biologically modified trichothecenes and zearalenone in food and the potential consequences of exposure on human health.

## 2. Mycotoxin Metabolism

Since plants are able to change the chemical structure of numerous masked mycotoxins, the toxicity of the latter may increase. Since modified mycotoxins are usually not detected by analytical methods commonly used to determine mycotoxins, their impact on food safety may be even more serious than it seems to be. The toxicology of these substances needs to be studied in greater detail. For these reasons, there are no established legal regulations related to the admissible levels of such mycotoxins in food.

The metabolism (biotransformation) of xenobiotics in plants may be divided into three phases [16]. During the first one, referred to as the *transformation phase*, some reactive groups are introduced into toxic molecules, for example via oxidation and/or reduction reactions using esterases, amidases, and the P-450 cytochrome system. In many cases, the activation of molecules in phase I of biotransformation is required for conjugation, but if reactive groups are already present in the parent molecule, such molecules may be conjugated directly. In phase II, also known as the conjugation phase, xenobiotics are conjugated with polar substances (often with glucose, sulphates, glutathione, or products of their degradation). Conjugation is a well-known detoxification strategy. The metabolism of xenobiotics in plants is assisted by the participation of plant enzymes, i.e., glucosyltransferases attaching a molecule, such as glucose, to the toxin and increasing its water solubility. Such compounds are transported to vacuoles for further storage or accumulated in the apoplast (phase III) [17,18]. Current knowledge suggests that mycotoxin metabolism in cereal plants applies mainly to toxins of the *Fusarium* fungi. The metabolites obtained are chemically varied compounds generated in enzymatically catalysed reactions [15].

**Figure 1:**
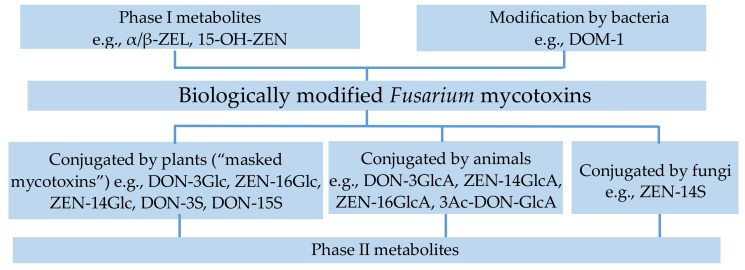
Taxonomy of biologically modified *Fusarium* mycotoxins.

The taxonomy of biologically modified *Fusarium* mycotoxins according to Rychlik et al. [12] is shown in Figure 1. 

### 2.1. DON and Its Analogues

The first mention of the natural occurrence of trichothecene glycosides in maize was reported in 1985 [19]. The metabolism of deoxynivalenol (DON) is best understood in plants. Amongst all DON metabolites, DON-3-glucoside (DON-3Glc) is the dominant product of the DON detoxification reaction, characterised by lower toxicity to plants than DON [20,21,22,23,24]. It was detected for the first time in 2005 as a contaminant of wheat and maize [25]. The presence of the DON-3Glc biosynthesis route was later confirmed in durum wheat and barley [26,27]. Amongst all modified mycotoxins, this is the most frequently detected compound in contaminated wheat grain [28]. Its co-existence with DON is a potential human health hazard, as this form may be hydrolysed in the gastrointestinal tract and lead to increased DON levels [29,30,31]. Other products of DON biotransformation include DON derivatives with glutathione (DON-GSH) and the products of glutathione degradation, i.e., DON-S-cysteinyl-glycine (DON-S-cys-gly) and DON-S-cysteine (DON-S-cys) [32,33,34].

**Figure 2:**
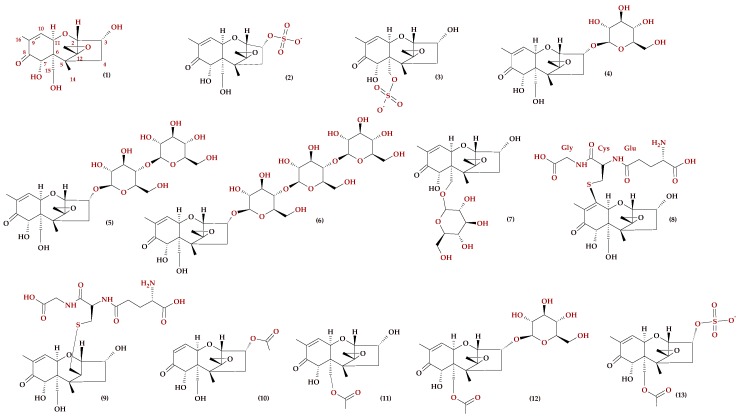
Chemical structure of some deoxynivalenol derivatives: (**1**) DON, (**2**) DON-3S, (**3**) DON-15S, (**4**) DON-3Glc, (**5**) DON-diG, (**6**) DON-triG, (**7**) DON-15G, (**8**) DON-10GSH, (**9**) DON-13GSH, (**10**) 3Ac-DON, (**11**) 15Ac-DON, (**12**) 15Ac-DON-3Glc, (**13**) 15Ac-DON-3S.

It is also appropriate to mention the first report on the potential biosynthesis of DON-3Glc from DON in the animal kingdom, by the English grain aphid (*Sitobion avenae*), which co-exists with a DON-producing organism (*F. graminearum*) during development of *Fusarium* Head Blight (FHB) in wheat [35]. In addition, a correlation between the susceptibility of field grain to FHB and the DON-3Glc/DON ratio in agricultural crop was found [36]. Recent years have identified new DON metabolites with biosynthetic routes based on the conjugation of DON with sulphates. Deoxynivalenol-3-sulphate (DON-3S) and deoxynivalenol-15-sulphate (DON-15S) have been isolated from wheat plants after artificial infection with *F. graminearum* and after direct injection into the ear 96 h after treatment [37]. However, other products of DON metabolism were detected after the artificial injection of DON into a wheat ear, in addition to DON-3Glc, DON-GSH, DON-S-cys-gly, and DON-S-cys, indicating the presence of DON-di-hexoside, 15-acetyl-DON-3-glucoside, DON-malonylglucoside, and ‘DON-2H’-S-glutathione [38]. The analysis of naturally contaminated wheat and oat grain containing more than 1 mg/kg DON confirmed the presence of metabolites such as DON-GSH, DON-S-cys-gly, DON-S-cys, and deoxynivalenol-*N*-acetylcysteine (DON-NAC), formed by a bond between the NAC group and the C-13 atom of the DON epoxy group [39]. The development of more sensitive mass spectrometry techniques has contributed to the discovery of new mycotoxin metabolites. Tests performed on the suspension cultures of wheat, with media supplemented with 3-acetyl-deoxynivalenol (3-AcDON), 15-acetyldeoxynivalenol (15-AcDON), 3,15-diacetyl-deoxynivalenol (3,15-di-AcDON), and DON, showed that deoxynivalenol-15-*O*-β-d-glucoside (DON-15Glc) and 15-acetyl-deoxynivalenol sulphate (15-AcDON-3S) may be synthesised in addition to the main metabolite of DON-3Glc. The possibility of the metabolism of 15Ac-DON to deoxynivalenol-3-*O*-β-d-glucoside (DON-3Glc) was also demonstrated. Equivalent processes with 3Ac-DON and 3,15-diAc-DON have not been confirmed, most probably because the acetyl group of 3Ac-DON blocks the C3-OH group [40]. DON and some of its metabolites are shown in Figure 2.

### 2.2. HT-2 and T-2 Toxins and Their Metabolites

**Figure 3:**
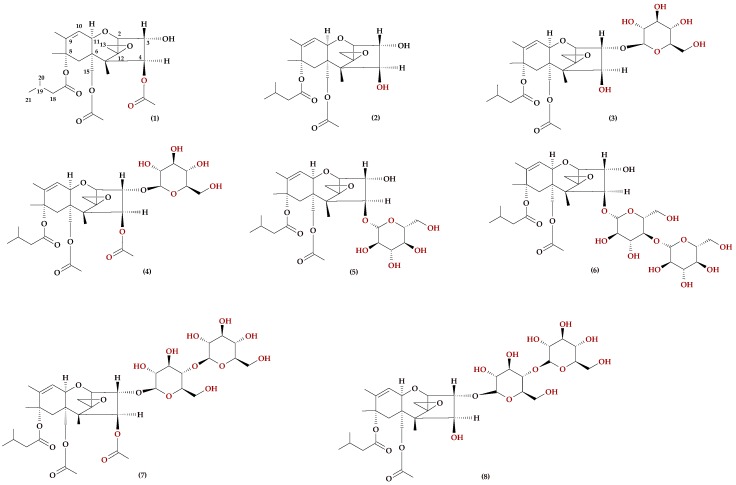
Chemical structure of some derivatives of HT-2 and T-2 toxins: (**1**) T-2 toxin, (**2**) HT-2 toxin, (**3**) HT-2-3Glc, (**4**) T-2-3Glc, (**5**) HT-2-4Glc, (**6**) HT-2-4diGlc, (**7**) T-2-3diGlc, (**8**) HT-2-3diGlc.

Similar to the biosynthesis of DON glycosides, potential metabolic pathways have been shown for HT-2 and T-2 toxins. These compounds are usually (and most abundantly) detected in oats. One of the first reports, published by Busman et al. [41], suggested that oat and wheat plants may metabolise HT-2 and T-2 toxins to T-2 3-*O*-glucoside (T-2Glc) and to HT-2-*O*-3-glucoside (HT-2Glc). The conclusions were reached after the mass spectrometric analysis of wheat and oat grain in artificially inoculated *F. sporotrichiodes*. The presence of glucoside HT-2 and T-2 toxins was also confirmed in naturally contaminated wheat, oat, maize, and barley grain [42,43,44,45,46]. It should be mentioned that glucoside conjugates of the T2 toxin have two anomer forms (most likely with different physical properties), but their relative toxicity is unknown [47]. Owing to the use of highly sensitive, high-resolution mass spectrometry in studies on the metabolism of mycotoxins, HT-2-di-glucoside (HT-2di-Glc) was detected in barley [42]. Subsequent studies also confirmed the presence of di-glucosides T-2 and HT-2 (T-2di-Glc, and HT-2di-Glc) in maize [45]. Mono- and tri-glucoside derivatives of T-2 and HT-2 toxins have also been isolated from maize plants and characterised by using high-resolution mass spectrometry. HT-2 and T-2 toxins, and their derivatives, are presented in Figure 3. The so-called “torn leaf” plant model was used to synthesise these conjugates in vitro [48]. The ability to transform the T-2 toxin into its glycoside forms has been confirmed not only in plants, but also in yeasts of the *Trichomonascus* species and the anamorphic species of the genus *Blastobotrys*. These species showed three biotransformation routes for the T-2 toxin: acetylation to 3-acetyl-T-2; glycosylation; and the removal of an isovaleryl group from the molecule, leading to the formation of neosolaniol [49]. Subsequent studies on metabolites of the T-2 toxin showed that the naturally present T-2-glucoside has an α-anomeric saccharide and that T-2-α-glucoside may be formed by *Blastobotrys muscicola* in amounts adequate for toxicity studies on animals [47]. It was also shown that *F. sporotrichioides* was able to form low levels of the glycoside derivatives of HT-2 and T-2 toxins [41,43]. Similar transformations were observed in *F. langsethiae*, in which the levels of the glycoside forms of the toxins were as high as 37% of that of the unconjugated toxin [46]. The detailed characteristics of T-2 and HT-2 toxin metabolism were presented by Nathanail et al., who showed the kinetics of the detoxification reaction pathways in spring wheat cv. Remus. Changes were observed over time, after the introduction of toxins to flowering wheat ears. This experiment produced the first documented presence of metabolites, such as HT-2-malonyl-glucoside, hydroxy-HT-2-glucoside, dehydro-HT-2-glucoside, T2-triol-glucoside, and T-2-feruloyl-T-2 toxin. Moreover, the process was shown to be extremely rapid, leading to an almost complete degradation of the toxin within one week after the studied substances were introduced to the flowering wheat ears [50]. A similar experiment performed on barley achieved at least partial identification of nine metabolites of the HT-2 toxin and 12 metabolites of the T-2 toxin. The metabolic pathways of these toxins were based on the hydrolysis of acetyl and isovaleryl groups, as well as on the hydroxylation and covalent binding to molecules of glucose, malonic acid, acetic acid, and ferulic acid. Moreover, the T-2 toxin was quickly metabolised to the HT-2 toxin, and HT-2-3-*O*-β-glucoside was the main metabolite identified for both toxins, with the maximum intensity of biosynthesis reached at 24 h after the toxins were introduced into the ear [51]. In the case of oats, studies on the metabolism of HT-2 and T-2 toxins led to the identification of 16 metabolites of the HT-2 toxin and 17 metabolites of the T-2 toxin. The flowering ears of oat were treated with toxins and the detoxification process was studied over time. The obtained derivatives resulted from the metabolic processes in phases I and II, including the hydrolysis reactions of ester groups, glycosylation, and hydroxylation reactions. Six of these metabolites (HT-2 hexosyl- and malonylglucoside hydroxy-derivatives) were confirmed with a high probability and described for the first time. However, similar to the other plant species, HT-2-3-*O*-β-glucoside was deemed to be the main metabolic product for the HT-2 and T-2 toxin, rapidly biosynthesised, and potentially undergoing further metabolic processes. The sum of the HT-2, T-2, and HT-2-3-*O*-β-d-Glc toxins comprised 88–106% of the total HT-2 toxin introduced into the ear; for the T-2 toxin, this value reached 81–100%. Other derivatives accounted for a maximum of several percent of the content of parent toxins [52].

### 2.3. ZEN and Its Metabolites

Zearalenone (ZEN) and its modified phase I metabolites, i.e., α-zearalenol and β-zearalenol (α-ZEL and β-ZEL) are contaminants of plants, cereal grain, and other products [53]. Their toxicity arises from their chemical structure, which allows coupling to the oestrogen receptor and exerts a strong influence on the reproductive systems of numerous animal species [54]. It was previously shown that ZEN can be transformed into its glycoside form in plant cells. In a suspension culture of maize cells, it was found that 13% of the initial ZEN content was zearalenone-4-*O*-β-glucoside (described as zearalenone-14-*O*-β-glucoside in recent literature, ZEN-14Glc) [55]. Naturally occurring ZEN-14Glc was also found in the edible parts of infected plants [56]. Amongst 24 samples of wheat grain, 22 contained 11–860 µg/kg ZEN, whereas ZEN-14Glc (17–104 µg/kg) was determined in 10 samples [57]. A breakthrough in studies on zearalenone metabolism in plants was connected with the first detection, in 2006, of the UDP glucosyltransferase gene, which encodes an enzyme responsible for the conversion of ZEN into ZEN-14Glc in *Arabidopsis thaliana* plants (UGT73C6 and AT2G36790). Yeast cells used in the genetic modification proved to be highly useful in the biosynthesis of this glucoside [58]. The same gene was used in the biosynthesis of α- and β-zearalenol-14-*O*-β-glycosides [59,60]. *Arabidopsis* seedlings are able to metabolise ZEN to ZEN-14Glc very rapidly. However, the accumulation of this glycoside is only periodic and further generated derivatives include complex compounds, e.g., zearalenone-malonyl-glucoside (ZEN-MalGlc), zearalenone-di-hexoside (ZEN-di-hexoside), zearalenone-pentosylhexoside [61]. Subsequent studies resulted in the identification of barley UDP-glucosyltransferase, HvUGT14077, able to transform ZEN into its modified form, into an almost equimolar amount of ZEN-14Glc and to small amounts of a new conjugate, zearalenone-16-*O*-β-glucoside (ZEN-16Glc). The presence of ZEN-14Glc and ZEN-16Glc was also found in barley seedlings, suspension cultures of wheat and *Brachypodium distachyon* cells, and barley seedlings. The levels of ZEN-16Glc were 18 times higher than that of ZEN-14Glc in barley roots. Moreover, the ZEN-16Glc conjugate may also be hydrolysed by intestinal bacteria that can transform the glucoside back to its free form [62]. UDP-glucosyltransferase shows a high affinity not only to ZEN, but also to its phase I metabolites, leading to the in vivo biosynthesis of phase II metabolites. Currently, studies on the biological functions of this enzyme and its suitability in commercial applications are in progress. The incubation of glucosyltransferase, the recombinant HvUGT14077 gene, and β-glucosidase produced up to 85% more ZEN-16Glc. Moreover, these studies indicated prospects for the application of this enzyme in the synthesis of other modified ZEN forms and α-ZEL and β-ZEL, both as mono- and di-glycosides [63]. Microorganisms, such as fungi, are also able to metabolise ZEN. In the 1990s, it was determined that the cultures of *F. graminearum* on inoculated rice showed the ability of these organisms to biosynthesise ZEN to zearalenone sulphate (ZEN-14-sulphate, ZEN-14S) through their metabolic pathways [64]. This form (ZEN-14S) is common contaminant of cereals and their derived food and feed products [65,66]. Studies performed by González Pereyra et al. showed that an in vitro culture of *F. graminearum* Z3639 on rice resulted in the biosynthesis of ZEN as well as phase I and II metabolites (α-ZEL and β-ZEL) and ZEN-14-sulphate [67]. *Rhizopus arrhizus* (IFO-6155) also displayed the ability to biotransform ZEN into its sulphate derivatives [68]. Subsequent studies showed that *Aspergillus niger* was also able to transform ZEN to ZEN-14-sulphate after incubation for 72 h with a wide range of ZEN concentrations in the substrate (5–150 µg/mL) [69]. The latter reports underline the importance of the fungal genera *Aspergillus* and *Rhizopus*. Two strains of *Aspergillus oryzae* and seven *Rhizopus* species displayed the ability to biotransform ZEN to various metabolites, such as ZEN-14S, ZEN-14Glc and ZEN-16Glc, and to phase I metabolites, such as α-ZEL and β-ZEL. These studies led to the isolation of α-zearalenol sulphate (α-ZEL-sulphate) [70]. Currently, no scientific reports have confirmed that ZEN-14S may be biosynthesised by cereal plants; *A. thaliana* is the only plant confirmed to be capable of such a transformation [71]. However, the fermentation processes using fungal organisms in food production may lead to the formation of various metabolic ZEN products in various detoxification routes [70]. In contrast, lactic acid bacteria (LAB) microorganisms used in the production of silage for animals were not able to biotransform metabolites produced by *F. graminearum* Z3639.

**Figure 4:**
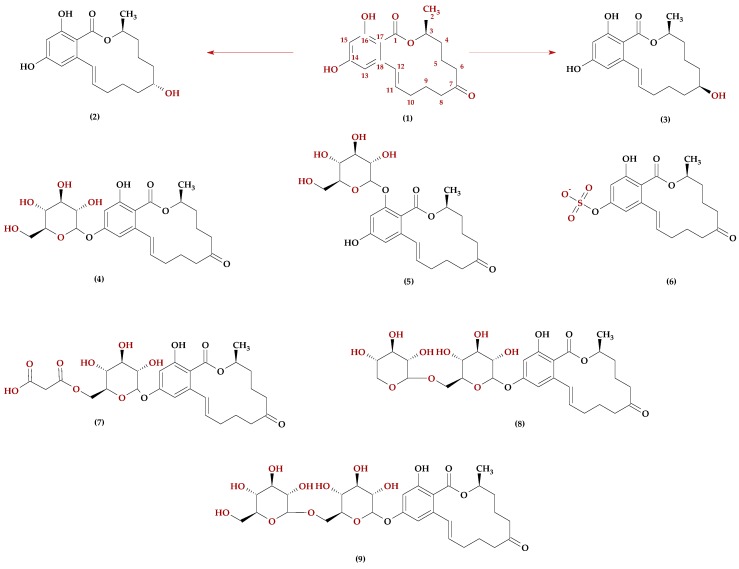
Chemical structure of some zearalenone derivatives: (**1**) α-ZEL, (**2**) ZEN, (**3**) β-ZEL, (**4**) ZEN-14Glc, (**5**) ZEN-16Glc, (**6**) ZEN-14S, (**7**) ZEN-MalGlc, (**8**) ZEN-14GlcXyl, (**9**) ZEN-14diGlc.

Modified ZEN derivatives, phase II metabolites, include β-ZEL-4-glucoside (β-ZEL-4Glc), which may be formed by maize during xenobiotic detoxification [67]. In the gastrointestinal tract, these conjugates may be readily hydrolysed to the parent mycotoxin [15]. ZEN and its derivatives are presented in Figure 4.

### 2.4. Metabolism of Mycotoxins in Animals

Two phases of metabolic detoxification have been identified in the animal kingdom. A known example of phase I metabolites is the deepoxidation of DON to deepoxy-deoxynivalenol (DOM-1), a route found in cows, pigs, and other animals. This type of metabolism is common in most studied animal species and occurs mainly with the participation of intestinal microflora [72,73]. Another equally notorious example of reaction products of the phase I mycotoxin metabolism is exo-8,9-epoxide of aflatoxin B1, obtained from aflatoxin B1, which is a toxin of *Aspergillus* spp. and considered to be a health hazard. This form results from oxidation reactions, including epoxidation, hydration, hydroxylation, and *O*-demethylation using the P-450 cytochrome (CYP-450), which occur mainly in the liver [74]. Several phase II biotransformation reactions have also been described, in which DON is conjugated with glucuronides, sulphonates, or glutathione [75]. The process of glucuronidation is involves UDP-glucuronyltransferases and may take place in intestinal microsomes [73]. Epoxidation is not particularly significant in humans and pigs, whereas glucuronidation is an important element of DON metabolism [29,76]. Mycotoxin conjugation reactions in animal bodies contribute to the formation of DON-3,8,15-glucuronides and HT2-3,4-glucuronides, which were also described recently [77,78]. Glucuronidation, de-epoxidation, and sulphonation were observed in poultry; however, the intensity of these transformations was low. Sulphation was discovered recently in chickens and turkeys as a metabolic pathway leading to the formation of the dominant metabolic product, i.e., DON-α-sulphate from DON [79,80,81]. Enzyme-catalysed glucuronidation is a slow process strongly dependent on the animal species [82].

## 3. Occurrence

As mentioned in the introduction, the natural occurrence of modified mycotoxins in cereal grains is investigated by using plant enzymes from substances produced by the enzymatic system of fungi, related to fungal infections of plants. DON and ZEN are biosynthesised mainly by *F. graminearum* and *F. culmorum*, which are mainly responsible for FHB [83,84,85], although *F. avenaceum* may also be involved. It is thought that the geographic distribution of *F. graminearum* and *F. culmorum* may depend on weather conditions, including temperature and relative humidity. *F. graminearum* is found mainly in warmer and humid areas (e.g., in North America, Eastern Europe, Eastern Australia, and in the south of China), whereas *F. culmorum* is mainly present in colder climates such as Western Europe [86]. This is not an absolute rule; in some geographic areas, the main FHB pathogens include *F. culmorum*, *F. avanaceum*, and *F. poae* [87]. Although DON and ZEN may be biosynthesised by *F. graminearum* and *F. culmorum*, the environmental conditions for each synthesis are different. For example, the most intense DON biosynthesis by *F. graminearum* occurs in acidic conditions (approximately pH 5.0) at approximately 24 °C, whereas ZEN synthesis requires a higher pH (approximately 7.0) and lower temperature (approximately 18 °C) [85]. A higher intensity of FHB is observed when cereal plants are exposed to long periods of high humidity, which potentially leads to an increase in mycotoxin biosynthesis [88,89]. The growth of *Fusarium* and the biosynthesis of DON also depend on the stage of plant growth. Wheat plants were more extensively damaged during the flowering stage and immediately. However, plants may also be infected during grain development [86,90].

The HT-2 toxin and T-2 are two related compounds that may be synthesised by several *Fusarium* species, mainly *F. langsethiae*, *F. sporotrichioides*, and *F. poae* [91,92]. Their presence in cereal grain has been well documented, with numerous reports originating in the cold, moderate climate of Northern Europe [93,94,95,96]. A limited number of reports indicated that the optimal conditions of water activity and temperature for the biosynthesis of HT-2 and T-2 were 0.980–0.995 and 20–30 °C [97,98]). A study by Kokken et al. showed that a water activity of 0.994 and a temperature of 15 °C promoted biosynthesis of these compounds by *F. langsethiae* and *F. sporotrichioides* [99]. *F. langsethiae* is more commonly detected in oat plants than in other cereal plants, which may explain the more frequent occurrence of HT-2 and T-2 toxins in this grain [100]. These compounds are toxic to most animal species in addition to humans. The toxic effects include protein synthesis and haematopoiesis inhibition, lymphoid depletion, and necrotic lesions [101].

Ecologic, agronomical, and genetic factors may contribute to the development of FHB and, consequently, mycotoxin accumulation. This requires an effective cereal crop management strategy. Fungicides do not provide full control over or prevention of mycotoxin presence. In addition, because of their harmful effect on human health and the environment, there is a tendency to significantly limit their use. These problems may be partially solved by farming programmes aimed at the improvement of plant resistance to *Fusarium* infections [102]. This resistance depends on the ability of the plant to prevent primary infections and the growth of toxic fungi; whereas resistance to mycotoxin contamination is also related to the ability of plant tissues to limit accumulation of mycotoxins [103]. This ability may arise from two mechanisms: the interruption of mycotoxin biosynthesis by host metabolites and the metabolic transformations of the toxin into less toxic compounds [104]. At present, no method to completely eliminate these mycotoxins from food is available and the current detoxification mechanisms contribute to the formation of many modified mycotoxins. Technological processes can actually affect modified mycotoxins by conjugation with other molecules of the matrices (e.g., sugars, proteins, or lipids) or the release of parent mycotoxins from their modified forms [105]. It should be noted that some cereal-derived products, e.g., beer produced from barley malt, may contain glucosylated mycotoxins [27,106,107]. During the beer production, an increased level of DON-3Glc in malt and beer was recorded in comparison with its concentration in barley. This results from the activation of vital functions during grain malting, in which the enzymes are able to transform mycotoxins into new metabolites such as DON-3Glc [108]. In addition, differences in the beer production process, alcohol content, and the quality of raw materials are also factors that influence the levels of mycotoxins (free and modified) in beer [27].

Similar situations are observed in thermal food processing, which influences the potential inactivation of mycotoxins [109,110,111,112]. Generotti et al. [105] investigated the effect of technological parameters (pH, temperature, mycotoxin level, and baking time) on the level of trichothecene in the raw material (whole grain) and cocoa biscuits. The increase in pH value and time during the baking phase effectively reduced DON and DON-3Glc content in the final products. In another experiment, the effects of milling and baking technologies on the levels of DON and its modified form (DON-3Glc) was determined in wheat samples grown under organic and conventional conditions [113]. In these experiments, the levels of mycotoxins (DON and DON-3Glc) were analysed in wheat milling fractions (white flour and bran), intermediate dough samples (kneaded, fermented, and proofed dough), and the final product (bread). The fractions of DON and DON-3Glc were similar in white flour and bran compared with their content in unprocessed grain. During the dough preparation process, no significant changes in the level of toxins were recorded, whereas baking resulted in a reduction in the content of both DON and DON-3Glc. Similar experiments on the fate of DON and DON-3Glc during the bread baking process at different temperatures (170–210 °C) and baking times (45–135 min) were conducted by Vidal et al. [114]. The DON level in bread was significantly lower than its initial concentration, but the DON-3Glc content was increased during kneading, fermentation, and baking.

Enzymes also play an important role in the baking process and can act directly on the DON-3Glc molecule, reconverting it to the parent mycotoxin DON. The addition of enzymes such as xylanase or α-amylase during the baking process resulted in an increased DON. The use of cellulases, proteases, and glucose oxidases can also promote an increase in the concentration of DON at the fermentation stage [114].

The data in Table 1 describe the occurrence of plant metabolites of mycotoxins identified in cereal grain and in some food products. To be able to relate the amount of mycotoxins to the amount of their modified derivatives, only results including at least one modified form have been taken into account. The majority of the data on DON, ZEN, HT-2, T-2 toxins and their modified forms compiled in Table 1 come from studies on cereal grains; only a small part come from research on cereal products or animal feed.

DON was found in the majority of analysed samples at concentrations within the LOQ (limit of quantification)–36,720 µg/kg range depending on the cereal/product type, and grain destination (food/animal feed). Concentrations of 3Ac-DON (range LOQ–2720 µg/kg) and 15Ac-DON (range LOQ–1730 µg/kg) were much lower. Also, the rates of occurrence of the latter derivatives were lower (see Table 1). DON-3Glc was found in a larger number of samples at somewhat higher concentrations, range LOQ–6600 µg/kg. Other modified forms of DON (e.g., its sulphate derivatives) are scarcely reported.

Data on ZEN and its metabolites are much less abundant than data on DON and its metabolites. ZEN concentrations ranged from LOQ to 15,700 µg/kg range; ZEN-14S concentrations ranged from LOQ to 4318 µg/kg. Concentrations of other modified forms of ZEN (ZEN-14Glc, ZEN-16Glc, α-ZEL, α-ZEL-14Glc, β-ZEL, β-ZEL-14Glc) were within the LOQ–283 µg/kg range.

Possibly toxic derivatives of the DON/ZEN/HT-2/T2 toxins in cereal grains may increase the risk of exposure of humans and animals to toxins. European regulations concerning maximum permissible levels of major mycotoxins in food depend on the foodstuff (DON: 200–1750 µg/kg, ZEN: 20–400 µg/kg), in feed also on animal age (DON: 900–12,000 µg/kg, ZEN: 100–3000 µg/kg) [10,115]. From the above limits and the data presented in Table 1, it seems that the risk of exposure of humans and animals to modified mycotoxins may be substantial and that efforts to extend the regulations to include in the future also DON/ZEN analogues are worthwhile. Still, more scientific reports on co-occurrence of DON/ZEN and their derivatives are needed to properly assess the severity of the issue.

Just a few reports on HT-2/T-2 toxins and their derivatives in cereal grains/products are available. The reported concentrations ranged from LOQ to 1830 µg/kg and from 1 to 377 µg/kg for HT-2 and T-2, respectively. Concentration data on HT-2-Glc and T-2-Glc derivatives are even scarcer since analytical-grade standards of these substances are quite difficult to obtain commercially; the reported ranges were LOQ–300 µg/kg and 0.1–14.5 µg/kg for HT-2-Glc and T-2-Glc, respectively. Comparing these figures with European MPLs for the sum of HT-2 + T-2 toxins (15–1000 µg/kg in food depending on the foodstuff, and 50–2000 µg/kg in forage depending on the feed target) one may not exclude a possibility that HT-2 and T-2 glucosides might significantly increase risk of exposure of humans and animals to the toxins [11]. To get more data on the co-occurrence of HT-2/T-2 toxins and their HT-2-Glc/T-2-Glc derivatives in cereal grains is therefore a serious challenge for researchers.

It must be concluded that more research on modified forms of mycotoxins in cereal grains/products is still needed. New ways to produce analytical-grade standards of these substances should be looked for if the research is to produce reliable results. The potential toxicity of modified mycotoxins is a very important topic from the food safety point of view. Further studies on natural occurrence and toxicology are also needed to be able to set future regulations concerning acceptable levels of free and modified mycotoxins. A need for such new regulations has been also been expressed by the scientific panel of the European Food Safety Authority [116].

## 4. Toxicological Properties

For a time, masked mycotoxins were thought to be a uniform group of compounds with lower toxicity than their parent analogues. In contrast, most recent studies have shown that masked mycotoxins are not functionally uniform and their safety/toxicity largely depends on the toxin type and the exposure [134]. Regulations and recommendations for the maximum permitted level of some mycotoxins (e.g., DON, ZEN, OTA, fumonisins, and others) have been introduced in many countries (in Europe: no. EU, 1881/2006 [10], with later changes). As the regulatory controls apply to the parent compounds (compounds selected to represent various chemical classes), they do not include the many modified forms that are commonly present in food and feed. This is mainly a result of the deficits of past toxicological studies related to modified mycotoxins, as legal regulations must be based on confirmed scientific data. The scientific community has now undertaken activities aimed at the discovery of the full metabolic profile of these forms in order to determine the influence of modified mycotoxins on human health [116]. Modified mycotoxins are a nascent problem in the scientific community, as no data are currently available on the toxicity of these modified toxins in vivo. Information on the genotoxicity and carcinogenic properties is still scarce. The shortage of such studies often results from the limited availability of pure compounds that can be used in toxicological studies. However, the development of potential routes leading to such substances undertaken by the scientific community should be mentioned. Such notable activities include chemical and biochemical synthesis using microorganisms and plants [47,48,120,135,136,137]. The limited accessibility and high complexity of in vivo studies create a barrier that prevents scientists from collecting information on the health effects of the metabolism of modified mycotoxins. Thus, other routes have been explored to determine the toxicological profile of these compounds. One such route includes testing on animals and simulated conditions in vitro digestion, which facilitates studies on the direction of the biotransformations of modified mycotoxins arising from the interaction of these substances with gastric acid and the content of the small intestine, as well as contact with the human microflora in the large intestine. Studies on animals create ethical complications. However, in vitro studies are difficult owing to the need for recreation of the complex processes of food digestion that occur in the individual parts of the human gastrointestinal tract, including biochemical reactions involving enzymes and intestinal microflora.

### 4.1. In Vitro Studies on DON and Its Derivatives

DON is a virulence agent of key importance in the increase of *Fusarium* wilt after the initial infection in cereal plants [18,138]. This compound is classified as a trichothecene, which belongs to a group of compounds known for their ability to inhibit protein synthesis [135]. *Fusarium* fungi producing DON may be divided into two chemotype sub-groups: 3Ac-DON and 15-AcDON, which may generate acetylated derivatives in addition to DON. The occurrence of these chemotypes may vary in different regions [139,140]. In 2010, the Expert Committee of FAO/WHO for food additives decided that the highest provisional maximum tolerable daily intake (PMTDI) determined for DON should also apply to acetyl derivatives of DON. This decision was made based on the knowledge that these compounds undergo de-acetylation in the body. Thus, it was decided that the health risk posed by the presence of these substances in the human gastrointestinal tract was potentially equal to the risk related to DON presence [141]. In 2017 the European Food Safety Authority issued scientific advice on hazards for people and animals brought about by DON and its acetylated/modified derivatives in food and feed. Cereals and cereal-based products were identified in the report as the main risk source. Just like for acetylated derivatives, toxicity of the DON-3Glc derivative was taken as equal to toxicity of DON itself because DON-3Glc in the intestine tract is readily hydrolysed to DON. On the basis of the epidemiological data collected so far, the acute group RfD for the compounds was set for 8 µg per kg bw [142]. Even if scientific community generally regards the compounds as toxicologically similar, studies performed in recent years have indicated that toxicities of DON/3Ac-DON/15Ac-DON are somewhat different. Recently performed studies on the use of the IPEC-1 cell line obtained from the small intestine of a newborn pig (in vitro), as well as using an intestinal transplant (ex vivo), and feeding piglets with feed contaminated with DON and its acetylated derivatives (in vivo) indicate potentially different toxicities for 3Ac-DON and 15Ac-DON. The intestinal epithelium is the first barrier for food contaminants and is highly sensitive to mycotoxins, particularly DON. These studies evaluated the influence of these substances on cell proliferation, barrier function, and the structure of intestines. The experiment showed that 3Ac-DON was less toxic than DON in the cell proliferation test; in turn, DON was less toxic than 15Ac-DON. 15Ac-DON reduced the protective functions of the intestinal epithelium, but 3Ac-DON and DON did not result in such changes. These observations were also confirmed in ex vivo and in vivo studies, in which histological changes to the intestines were more significant after 15Ac-DON treatment than after DON and 3Ac-DON treatment [143,144] and also indicated that the two acetylated derivatives may display different toxicities. Analysis of gene expression in the mutated *pdr5D* yeast strain exposed to mycotoxins revealed that 15Ac-DON induced changes in gene expression. The exposure of yeast cells to DON usually had a similar, but less profound, influence on gene expression. 3Ac-DON resulted in different changes in expression of these genes than DON and 15Ac-DON [144,145]. The in vitro cytotoxicity of modified DON forms towards differentiated epithelial cells from the intestines of newborn piglets and their proliferation was also determined. After a 72-h exposure of IPEC-2 cells to modified DON forms, the cytotoxicity of these forms was observed to follow the pattern DON-3Glc << 3Ac-DON < DON ≈ 15Ac-DON. These differences were attributable to the non-conjugated hydroxyl group at the C3 carbon atom, which has a significant role in the ability of the molecule to bind to ribosomes and influenced its potential toxicity [145]. These results also confirmed the study of Alizadeh et al., who used the human intestinal cell line Caco-2 to evaluate their viability and barrier integrity, and determined the release of the pro-inflammatory CXCL8 cytokine. 3Ac-DON proved to be less efficient for the induction of unfavourable changes in barrier integrity compared with DON, whereas 15Ac-DON appeared to be slightly more effective than DON. Moreover, DON and its derivatives induced the release of the pro-inflammatory CXCL8 cytokine in Caco-2 cells [146]. Similar results were obtained by de Loubresse et al. [147] and Wu et al. [148]. Thus, further studies are required to evaluate the toxicity of modified DON derivatives.

The fact that DON-3Glc may be hydrolysed in lower parts of the alimentary tract has been confirmed in several scientific reports. In recent years, Berthiller et al. [30] supported the toxicological importance of DON-3Glc through the confirmation that some lactic acid bacteria may hydrolyse DON-3Glc during in vitro digestion. To mimic the stages of digestion by employing acidic, enzymatic hydrolysis and stool fermentation using intestinal bacteria, it was shown that DON-3Glc was stable in 0.2 M HCl (pH 1.7) for at least 24 h at 37 °C, which suggested that these conjugates would not be hydrolysed in the mammalian stomach. Human cytosolic β-glucosidase also caused no changes in the structure of masked mycotoxins. Amongst the forty-seven different bacterial strains isolated from intestines, faecal bacteria and lactic acid bacteria, such as *Enterococcus durans*, *Enterococcus mundtii*, or *Lactobacillus plantarum*, may degrade DON-3Glc. Similar in vitro study was performed by Dall’Erta et al. [31] within artificial stomach/intestinal part of an alimentary tract. They demonstrated that DON-3Glc did not decompose after 30 min, although after 24 h it totally degraded. After that fermentation period, DON was the main decomposition product present in the faecal suspension (90%). Only trace amounts of the DOM-1 deepoxidation product were found in the post-fermentation mass [31]. Gratz et al. [149] confirmed the ability of faecal bacteria to hydrolyse DON-3Glc to DON owing to the production of hydrolytic enzymes capable of cleaving the bonds between glucosides and the toxin. These studies included tests on faeces obtained from five volunteers, which were incubated under anaerobic conditions with DON-3Glc. The analyses confirmed that DON-3Glc may be rapidly hydrolysed. The complete hydrolysis of DON-3Glc was observed in faeces obtained from four people within 6 h of incubation. In addition, the rapid hydrolysis of DON-3Glc to DON was observed in the faeces of one of the participants (up to 2 h incubation); this was followed by the presence of DOM-1 observed after incubation for 6 h. The presence of DOM-1 was not identified in material from other volunteers, even after seven days of incubation [149]. Similar results were reported by Gratz et al. [150], who studied hydrolysis of DON-3Glc in pig intestines. Samples of digested byproducts collected post-mortem from jejunum, ileum, cecum, colon, and faeces were incubated for 72 h with DON-3Glc or free DON in anaerobic conditions. Small intestine microflora hydrolysed DON-3Glc very slowly, while samples from ileum, cecum, colon, and faeces were hydrolysed quickly and efficiently. No further metabolism of DON was observed in any of the samples even if composition of microflora residing in ileum was quite different than composition of microflora in the distal gut, while composition of microflora in cecum, colon, and faeces did not differ [150]. 

Additionally, De Nijs et al. [151] used a static simulation model of in vitro digestion, mimicking the top section of the gastrointestinal tract (salivary glands and the stomach) and the bottom section of the gastrointestinal tract (the small intestine). The transformation of DON-3Glc in the small intestine was evaluated by using Caco-2 cells in the Transwell^®^ system. Those authors also concluded that DON was not released from DON-3Glc in the mammalian stomach and that it was resistant to human cytosolic β-glucosidase. One hypothesis proposed that the bioavailability of DON-3Glc may be lower than DON, as Caco-2 cells did not absorb DON-3Glc, unlike DON. Similarly, Alizadeh et al. concluded that DON-3Glc had no adverse influence on the integrity of cell membranes in the Caco-2 model in the Transwell^®^ system. DON-3Glc (as DOM-1) was proven to be significantly less active than DON and its acetylated forms for the induction of the CXCL8 chemokine [146]. Studies performed by De Angelis, Monaci, and Visconti confirmed the high stability of DON during the digestion of bread contaminated with DON and DON-3Glc in the stomach when evaluated under simulated digestion conditions. A decrease of 43% in DON levels was found in the passage from the stomach to the duodenum, unlike DON-3Glc, which was not significantly altered between the beginning and the end of digestion in the stomach. However, increased levels of DON-3Glc were observed after digestion in the duodenum. The authors suggested that the increase in DON-3Glc during food digestion, between leaving the stomach and the end of digestion in the duodenum, was a result of the interaction between DON and glucose units released as a result of the activity of enzymes present in the duodenum. The increase in DON-3Glc after the digestion of bread in the duodenum may also be explained by the presence of matrix interactions and/or by physical trapping within the matrix structure, which prevents mycotoxin detection using the standard method. The increased DON-3Glc content may therefore result from an interaction and release from the structure, caused by better dispersion of the digestive contents in the duodenum [152]. Potentially lower DON-3Glc toxicity was also proposed by Suzuki and Iwahashi, who used growth tests and the analysis of a DNA micromatrix of a mutated yeast strain *Saccharomyces cerevisiae* PDR5, exposed to DON-3Glc and found that DON-3Glc was less toxic towards yeast cells than DON [153]. The lower toxicity of DON-3Glc was also reported in a study by Pierron et al. Experiments on the ability to induce cell death via stress induction (so-called ribotoxic stress), as well as the induction of intestinal toxicity, were performed in Caco-2 cells (in vitro) and in ex vivo studies using explants from pig intestines. The results showed that in contrast to DON, DON-3Glc was unable to bind with the A-site of the ribosome peptidyl transferase centre. Explants exposed to DON-3Glc exhibited no histomorphological changes; thus, it was concluded that glycosylation inhibited the ability of DON to bind with ribosomes and decreased its intestinal toxicity [154]. However, DON-3Glc may pose a health hazard if it is hydrolysed to DON in the gastrointestinal tract [155]. Data related to the lower toxicity of DON-3Glc contrasted with the data of Tian et al., and provide hope that a method of crop protection against *F. graminearum* will be developed. In that study, eight strains of *Trichoderma* were selected in order to evaluate their antagonistic activity towards *F. graminearum* in a double culture test. Four of these strains were characterised by the unusual inhibition of both mycelium growth and mycotoxins produced by *Fusarium*. Moreover, it was shown that, in the presence of *F. graminearum*, *Trichoderma* was able to metabolise DON to DON-3Glc by using a protective mechanism [156].

Lower toxicity was also shown in vitro in the case of products of DON metabolism generated by bacteria (i.e., DOM-1 and 3-epi-deoxynibalenol (3-epi-DON)). These compounds were not toxic towards intestinal epithelium cells and did not impair the barrier function of the intestines. During ex vivo studies, no changes to explants exposed to these metabolites were observed. DON and its metabolites may be bound to the A-site of the ribosome peptidyl transferase centre, wherein three hydrogen bonds were formed with the A-site and activated mitogen-activated protein kinases (MAPK). Other compounds formed only two hydrogen bonds and did not activate MAPK, which contributed to the lower toxicity [72].

A number of relations that should be taken into account when assessing impact of DON and its derivatives on living organisms have been revealed in the course of the above mentioned research on toxicity of the compounds. The in vitro studies helped to model complex processes running during digestion and to identify paths along which various mycotoxin forms are bio-transformed. The paths depend on many factors, of which the microbiome composition in individual sections of the digestive tract is probably the most important one (the composition is very difficult to model in vitro as it is different for each individual organism). Other factors include absolute concentration of any given mycotoxin and its share in mycotoxin profile within the intestinal content, exposure history, dietary conditions etc. All that makes the task of reliably assessing a safe level of DON (with its derivatives taken into account) in food/feed a very difficult one.

### 4.2. In Vitro Studies on ZEN and Its Derivatives

In addition to DON, ZEN and its derivatives were also studied by Dall’Erta et al. [31], who found that modified forms of the mycotoxins were deconjugated by the microflora of the human colon. During an in vitro experiment involving the incubation of gastrointestinal contents containing mycotoxins, their toxic aglycones were released and unidentified catabolites were generated. The enormous variety of bacterial strains and the enzymes they produce in the human colon mean that the gastrointestinal tract can essentially be considered a huge bioreactor, where the chemical transformations of most compounds consumed by humans occur. The in vitro digestion test was performed by using ZEN-14Glc, ZEN-14S in four artificial digestive solutions: saliva, gastric acid, duodenum juice, and bile. After incubation in these juices, the modified mycotoxins were recovered at 99.5%, 97.3%, and 98.6%. An in vitro faecal fermentation test was simultaneously performed over periods of between 30 min and 24 h, to study the potential decomposition of modified mycotoxins. After 24 h, the total decomposition of ZEN-14Glc and ZEN-14S was observed. Moreover, ZEN was only partially recovered from faeces after fermentation, which showed that further ZEN degradation to unknown catabolites occurred in this part of the gastrointestinal tract. 

ZEN is metabolised in animal bodies, mainly to α-ZEL and β-ZEL. This process, which occurs in the liver, primarily involves 3-α/β hydroxysteroid dehydrogenase [157]. The relative ratio of these two ZEN metabolites depends on animal species. Studies using a model of the intestinal epithelium and the human Caco-2 cell line showed that the ratio of these two metabolites was 5:4 [158]. It was also shown that further reduction of α-ZEL and β-ZEL may lead to a significant increase in α-zearalanol (α-ZAL) and β-zearalanol (β-ZAL). α-ZAL is metabolised mainly to the diasteroisomer β-ZAL and, to a smaller extent, to zearalanone (ZAN). Subsequently, these metabolites are conjugated with glucuronic or sulphonic acid and excreted in urine [159]. The toxicological properties of products of phase I of detoxification have been studied over the last decade. They were suspected to be cytotoxic towards the Vero cell line [160] and towards immune cells [161], were found to have a significant effect on cell proliferation in the Jurkat C cell line [162], and exhibited genotoxic properties [163,164]. Despite numerous studies confirming the cytotoxic properties of ZEN and its phase I derivatives, the cytotoxicity of α-ZEL and β-ZEL appears to dependent on cell type. In recent years, attempts have been made to explain the toxicity levels of ZEN derivatives towards their basic analogue. Studies on cytotoxicity and genotoxicity of α-ZEL and β-ZEL were performed in mice bone marrow cells and human HeLa cells. The toxic effect of ZEN and its metabolites was evaluated separately in the induction of DNA damage by using a chromosomal aberration test in vivo (CA) (bone marrow cells of BALB/c mice) and in vitro (HeLa cells). The results indicated that the toxicities of ZEN and α-ZEL were the same and that both these compounds were more cytotoxic than β-ZEL. All three substances increased the rate of chromosomal mutations in murine bone marrow cells and in HeLa cells. In both systems, ZEN and α-ZEL induced the same extent of genotoxicity and both compounds were more toxic than β-ZEL [165]. The cytotoxicity of ZEN and its phase I metabolites have also been studied in ovarian cells of Chinese hamsters (CHO-K1). The cytotoxicity was examined in an MTT assay, which confirmed that α-ZEL was more cytotoxic than ZEN or β-ZEL towards the CHO-K1 cells [166,167]. The effect of ZEN and its metabolites (α-ZEL and β-ZEL) on the parameters of the epithelium in cell lines of the small intestines of newborn piglets (IPEC-1) have been studied. Parameters of key importance for the function of intestinal epithelium were studied, including cell viability, cytokine synthesis, and epithelium integrity. These experiments showed that ZEN metabolites were more toxic towards IPEC cells. ZEN had no effect on the value of trans-epithelial electric resistance (TER), whereas α-ZEL significantly decreased TER from the fourth day of the experiment. β-ZEL resulted in two effects: initially, it induced a significant increase in TER values, but from the sixth day of the experiment, it caused a significant decrease in TER values compared with the initial value. Moreover, ZEN showed the ability to amplify the synthesis of IL-8 and IL-10 cytokines, whereas α-ZEL and β-ZEL inhibited the expression of IL-8 and IL-10 cytokines as a function of their concentration. Thus, ZEN and its metabolites may the influence viability of pig intestine cells, the integrity of the intestinal barrier, and the synthesis of cytokines, which are all important from an intestinal health perspective [167]. Despite numerous studies, the mechanisms underlying the cytotoxic activity of ZEN derivatives remain unknown, although attempts continue to gain insight into these complicated processes. An experiment of Ben Salem et al. focused on the mechanism of toxicity induced by α-ZEL and β-ZEL in human HCT116 cells. It was shown that the presence of α-ZEL and β-ZEL generated stress in the endoplasmatic reticulum (ER) via the mitochondrion-dependent pathway, which manifested as a loss of transmembrane mitochondrial potential, further production of reactive oxygen species O_2_^•−^, and the activation of caspase-3. However, the presence of quercetin, a flavonoid found in the human diet, may mitigate the negative effects caused by α-ZEL and β-ZEL in the cells. Thus, it was concluded that quercetin conferred protective activities against the cytotoxic effects of α-ZEL and β-ZEL [168]. Drzymala et al. stressed the importance of *cis*-ZEN. Since trans-ZEN naturally biosynthesised by *Fusarium* may be transformed into *cis*-ZEN under the influence of UV light, that latter form may appear in food. In vitro experiments conducted on phase I metabolism of *cis*-ZEN in human and rat liver microsomes showed that the metabolic pathway of this form may be similar to that of *trans*-ZEN. α-*cis*-ZEL, produced during the metabolic pathway of *cis*-ZEL, may exhibit higher oestrogenicity than its isomer [169]. Although the estrogenic properties of ZEN are well described, much less is known on their interactions with other nuclear receptors. Frizzell et al. [170] analysed the potential effects of the disruption of hormones at the level of the nuclear transcription activity receptor caused by ZEN and its metabolites, α-ZEL and β-ZEL. Their influence on the endocrine system was studied by using gene tests containing natural steroid receptors (oestrogen, androgen, and progestagen) and in a steroidogenesis test by using the (steroidogenesis) H295R model. α-ZEL demonstrated the strongest estrogenic properties; the weakest estrogenic properties were shown by β-ZEL. Progesterone binding to the progestagen receptor was found to be synergistically increased in the presence of ZEN, α-ZEL, or β-ZEL. Moreover, each of these compounds increased, to a different extent, the synthesis of progesterone, estradiol, testosterone, and cortisol hormones in the steroidogenesis test, which confirmed their influence on the endocrine system [170]. In turn, Molina-Molina et al. studied the interactions of ZEN and its metabolites with five human androgen receptors (hAR) and the α type oestrogen receptor (hERα). ZEN and its reduced metabolites (α-ZEL, β-ZEL, α-ZAL, β-ZAL, and ZAN) were shown to be full hERα antagonists and all six tested compounds showed activity towards hAR. Thus, ZEN and its metabolites may disrupt the function of the human and animal endocrine systems through various mechanisms of action [171].

Oestrogen activity was also analysed for ZEN-14-*O*-glucuronide, α-ZEL-14-*O*-glucuronide, α-ZEL-7-*O*-glucuronide, β-ZEL-14-*O*-glucuronide, and β-ZEL-16-*O*-glucuronide in a gene test (RGA). As all studied compounds were characterised by similar activity losses, it was concluded that the reactions of ZEN and its phase I metabolites with glucuronic acid were detoxification reactions in terms of estrogenic properties. These reactions constitute a potential defensive mechanism of the body against estrogenic activity caused by ZEN [172]. A similar loss of the ability of ZEN-14Glc to bind with the oestrogen receptor was observed in other studies [58].

Reduced and glucuronided ZEN metabolites were for a long time the only known ZEN biotransformation products in mammals. Using human hepatic microsomes it was demonstrated that ZEN may be monohydroxylized at C13 and C14, producing 15-OH-ZEN, the main metabolite [173]. Research on estrogenicity of 15-OH-ZEN, ZEN-14S, α-*cis*-ZEL and β-*cis*-ZEL (using the E-Screen test) showed that the *trans-*to-*cis* change of configuration resulted in maintaining estrogenic activity, while the activity significantly decreased as compared to ZEN in other cases [174]. 

Just like in case of DON, toxicological studies of ZEN and its derivatives have shown extreme complexity of the processes along which they are bio-transformed. Various microorganisms residing in individual sections of the digestive tract certainly play an essential role. Since ZEN and its derivatives show strong oestrogenic action, their level is strongly influenced by steroid hormones capable to interact with the toxins. The so-far conducted research has also shown that a diet rich in flavonoid compounds may mitigate toxicity of ZEN and its metabolites.

### 4.3. In Vivo Studies on DON/ZEN/T-2 Derivatives

Health hazards caused by the presence of ZEN glycosides are based on their in vivo hydrolysis ability. Recent studies on the subject document the potential transformation of these toxins in the circulatory system. Such transformations may apply not only to ZEN-14Glc, but also to ZEN, a potential precursor of α-ZEL and β-ZEL [23,134,175]. In other studies, two modified forms of ZEN, ZEN-14Glc and ZEN-16Glc, were transferred to the intestine by using polarised Caco-2 cell monolayers to investigate the possible intestinal absorption of the glucosylated forms of ZEN. The cells were exposed to 20 and 40 mM ZEN-14Glc, ZEN-16Glc, and ZEN, separately [176]. The results showed that after apical administration, the two glucosylated forms (ZEN-14Glc and ZEN-16Glc) can be detected in cellular extracts, which indicated uptake by intestinal cells; however, ZEN-14Glc appeared to be more prone to deglycosylation than ZEN-16Glc.

**Figure 5:**
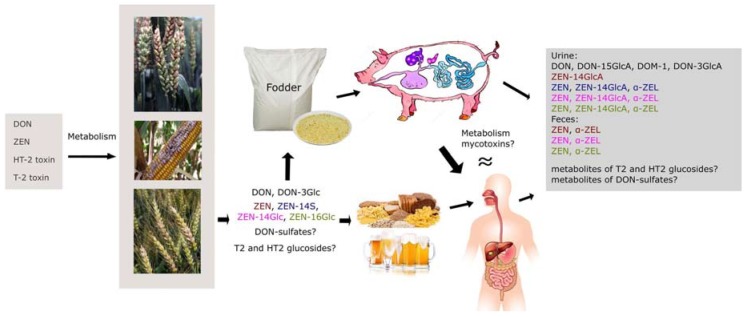
Mycotoxins and their modified forms in cereal plants and potential paths of their bio-transformation in humans (compiled from literature data).

As previously mentioned, unlike in vitro studies, in vivo studies are scarce because of the complexity and limited availability of appropriate amounts of reference compounds. At present, only a small number of tests on animals have been performed by using modified mycotoxins. The toxicological characteristics of modified mycotoxins are usually observed after the introduction of compounds into feed of animals with comparable anatomic structure to humans. In the case of the heart and the circulatory system, the kidneys, liver, and the gastrointestinal tract are thought to be most closely resembled by the pig anatomy [29]. Moreover, pigs are considered to be the most susceptible species to DON, a property related to their weak ability to metabolise DON to DOM-1 and rapid absorption of metabolised DON. For these reasons, pigs are considered to be the best animal model for in vivo studies [177]. Possible paths of transformations of modified mycotoxins in humans compiled from the literature data are shown in Figure 5. Some authors point out that metabolism of DON depends on concrete animal species, both in terms of the possible transformation paths and the process speed. Maul et al. [81] evaluated metabolism of DON in vitro using human microsomal fractions and uridine 5′-diphospho-glucuronosyltransferase (UDP-glucuronosyltransferase, UGT), and five fractions of hepatic microsomes from various animal species [81]. Such discrepancies show that further in vitro and in vivo studies are needed to get more insight into animal modelling of metabolism of various mycotoxins (including DON) in humans. 

In an experiment conducted by Nagl et al. [29], four piglets were provided with ad libitum access to drinking water and received DON-3Glc (116 mg/kg of bodily mass), together with equimolar amounts of DON (75 mg/kg of bodily mass) through a stomach tube on days 1, 5, and 9. Additionally, 15.5 mg DON-3Glc/kg of body mass were administered on day 13, after which the levels of DON, DON-3Glc, DON-3GlcA, deoxynivalenol-15-glucuronide, and DOM-1 were determined in the faeces and urine by using UHPLC-MS/MS. After the oral administration of DON and DON-3Glc, approximately 85% and 40% of the administered dose, respectively, was detected in urine. Only trace amounts of the metabolites were detected in the faeces. The data indicated that over 50% of the administered DON-3Glc was decomposed in the gastrointestinal system of piglets, despite the relative stability of DON after systemic absorption. Broekaert et al. confirmed that DON-3Glc may show lower toxicity than DON. DON-3Glc and DON were administered to broiler chickens and pigs both as food (in vivo) and intravenously and the DON metabolites were analysed by using liquid chromatography and a high resolution mass spectrometer. The results showed that DON-3Glc did not hydrolyse to DON in the gastrointestinal tract of the chickens and its bioavailability was comparable with that of DON. In contrast, in pigs, only DON was detected in the blood plasma, which indicated that DON-3Glc had been hydrolysed. However, the absorbed DON-3Glc fraction (recovered as DON), was approximately 5-fold lower than the DON concentration when absorbed from with food. Moreover, it was indicated that the biotransformation of DON and DON-3Glc in pigs occurred predominantly through glucuronidation, whereas in chickens this occurred through conjugation to a sulphate. In chickens, the formation process of phase II metabolites was more intense than in pigs, which may explain differences in the sensitivity of the toxicological effects of DON between these animal species [178]. DON-3-sulphate and DOM-3-sulphate were also the main metabolites in broiler chickens, hens, and turkeys, as confirmed by Schwartz-Zimmermann et al. They were formed after the birds were fed with feed supplemented with an equimolar mixture of DON and DOM-1 [179].

Toxicological studies on animals by using DON and its derivatives were also performed on rats. After the administration of the acetylated forms of DON (3- and 15Ac-DON) to rats, free DON was found in the stomach at 11.4% (3Ac-DON) and 15.5% (15Ac-DON). After the administration of DON-3Glc, DON was found at trace levels of <3%, confirmed by Berthiller et al. [30], who reported the stability of DON-3Glc in acidic media. The formation of the glucuronide Ac-DON was also described. Rats may directly glucuronise AcDONs without previous deacetylation. After the administration of 3Ac-DON and 15Ac-DON to rats in via food, glucuronised 3Ac-DON (3Ac-DON-GlcA) accumulated in the small intestine, together with DON-3GlcA [180,181], and it was found that DON-3Glc was partially bioavailable in rats. Six rats received water, DON (2 mg/kg of bodily mass) and an equimolar amount of DON-3Glc via a stomach tube, on days 1, 8, and 15 of the experiment. The urine and faeces were collected and analysed for DON, DON-3Glc, glucuronate (DON-GlcA), and DOM-1 by using LC-MS. Those studies confirmed that only 21% of administered DON-3Glc was found in the faeces, mostly as DON and DOM-1. It was also found that DON-3Glc was digested in the distal part of the intestine. However, the study did not explain the fate of the 79% of DON-3Glc that was not detected. Some authors still claim that the presence of this compound in food and feed appears to induce lower toxicity than DON [181].

Currently, very little data are available on the toxicity of modified ZEN forms in studies performed on animals in vivo. These data are missing for many animals species [182]. One of the reports was of Binder et al. [183], who studied the transformations of modified ZEN forms (ZEN-14Glc, ZEN-16Glc, and ZEN-14S) in pigs through the introduction of equimolar amounts of these metabolites in food. ZEN and its modified forms, including mammalian metabolites, i.e., ZEN-14-glucuronide, α-ZEL, and α-ZEL-14-glucuronide, were subsequently analysed in faeces. After the administration of ZEN to the gastrointestinal tract, 26 ± 10% of the original amount of this compound was recovered in urine and 14 ± 3% in faeces. ZEN-14S, ZEN-14Glc, and ZEN-16Glc were detected neither in the urine nor in the faeces. After the application of ZEN-14S, 19 ± 5% of the administered dose was found in urine, whereas no ZEN metabolites were detected in faeces. The total recovery of ZEN-14Glc and ZEN-16Glc in urine and faeces was 48 ± 7% and 34 ± 3%, respectively. These studies suggested that the low recovery rate for compounds introduced into the animal body was related to the existence of the metabolic pathways of intestinal bacteria, which lead to the formation of currently unknown metabolites. Moreover, all studied modified ZEN forms were fully hydrolysed in the gastrointestinal tract to ZEN. Thus, they contribute to the overall ZEN toxicity.

So far no in vitro research results on the toxicity of HT-2/T-2 toxin derivatives that might help us to understand the bio-transformation of the toxins have been published. The few results cited in Table 1 show that HT-2/T-2 glucosides were found in a large fraction of all tested samples, which means they might be an essential issue. Unfortunately, their toxicity in animals has been studied in vivo in just a few cases only. In recent years in vivo research on T-2 and T-2Glc in broiler chickens was reported [184]. It was demonstrated that T-2Glc was hydrolysed to the T-2 toxin to a negligible extent only.

Numerous authors have reported different toxicity of various DON-acetylated derivatives. The results of in vitro and in vivo animal studies concerning the toxicity of DON-3Glc have been mounting for several years; the majority of authors have reported the potentially lower toxicity of modified DON derivatives. However, the latter compounds are usually accompanied by DON, so the total exposure might be higher in spite of the somewhat reduced toxicity of the modified forms. Therefore, every scientific result on the co-existence in food of DON with its modified forms may be essential. This is particularly true as some new DON metabolites (like DON sulfate derivatives) have recently been found in cereal grains; unfortunately, no data on their toxicity are available.

Data on directions of biotransformation of modified ZEN derivatives in human and animal organisms are scarce, except for derivatives produced in reactions running during the first phase of xenobiotic detoxification: the highest estrogen activity was found for α-ZEL. Both in vitro and in vivo studies on pigs showed that conjugated ZEN forms (phase II of the detoxification reactions) most probably may be hydrolysed in the human gastrointestinal tract (although the intensity of the process might be different for different species), that way increasing ZEN toxicity. The reliability of health hazard evaluations is still limited in view of too few available scientific data on the occurrence of the toxins in food. The highest deficit both in terms of data on hydrolysis within the gastrointestinal tract as well as data on cytotoxicity is observed for HT-2/T-2 glucosides. Some results of studies on poultry suggest that they are not hydrolysed within the gastrointestinal tract, but the results need to be confirmed both in vitro and in vivo with respect to various other animal species.

Very little is known on the possible synergy between co-existing parent mycotoxins and their modified derivatives; that topic is a challenge for future researchers. A lack of analytical standards of those compounds (except commercially available DON-3Glc) is an issue in any research on modified mycotoxins produced during phase II of the detoxification reactions. That obstacle naturally hampers research on the occurrence of the compounds in food/feed, their toxicological profile, and the evaluation of food-safety-related risks. 

## Figures and Tables

**Table 1:** Natural occurrence of modified mycotoxins in cereals and cereal-based foods and feed.

Mycotoxin	Cereals/Cereals Based Food	Positive Samples	Min–Max [µg/kg]	References
DON	maize	6/6 (100%)	255–5245	[117]
		3/3 (100%)	90–680	[25]
		10/10 (100%)	32–2246	[118]
		9/10 (90%)	74–1382	[119]
		-	LOQ *–5660	[120]
		51–100%	0.3–4374	[121]
	maize silage for feed and ready feed	799/1113 (71.8%)	1.5–13,488	[122]
	maize products	91–97%	0.3–2803	[121]
	barley	28/34 (82.4%)	LOQ–1180	[123]
		15/15 (100%)	<60	[118]
		20/24 (83%)	54–1602	[124]
	wheat	76/92 (83%)	11–1265	[125]
		29/30 (97.6%)	LOQ–5510	[123]
		4/6 (66.7%)	16–150	[117]
		5/5 (100%)	540–5080	[25]
		6/6 (100%)	46–2683	[118]
		46/99 (46.5%)	25–2975	[124]
		5/5 (100%)	4680–36,720 **	[28]
		6/13 (46.1%)	LOQ–297	[126]
		15/20 (75%)	LOQ–10,130	[127]
	durum wheat	84/84 (100%)	1750 (average)	[128]
	triticale	4/5 (80.0%)	43–737	[118]
		20/20 (100%)	196–1326	[124]
	rye	12/12 (100.0%)	<50	[118]
	oat	31/31 (100%)	2690 (average)	[123]
		1/6 (16.7%)	46	[117]
		9/11 (81.8%)	62–2216	[118]
		4/4 (100%)	67–149	[124]
	bread	4/6 (66.7%)	20–102	[117]
	corn flakes	1/6 (16.7%)	207	
	white flour products	16/17 (94.1%)	5–30	[129]
	mixed flour and whole-corn products	32/36 (88.9%)	13–431	
	breakfast cereals	2/7 (28.6%)	31–347	
	snacks	21/34 (61.8%)	13–320	
	flours	16/22 (72.3%)	28–594	
DON	wheat flour	349/359 (97.2%)	1.3–825.9	[130]
		4/4 (100%)	78.9–325.8	[126]
		5/5 (100%)	940–5890 **	[28]
	feed for swine	15/16 (93.8%)	50–931	[119]
	feed for poultry	14/15 (93.3%)	157–1231	
	beer	18/20 (90%)	LOQ–35.9	[131]
	light beer	118/217 (54.4%)	<LOQ–89.3	[132]
	wheat beer	36/46 (78.3%)	<LOQ–49.6	
	dark beer	14/47 (29.8%)	<LOQ–45.0	
	bock beer	18/20 (90.0%)	LOQ–27.1	
	alcohol free beer	5/19 (26.3%)	LOQ–26.1	
	shandy beer	13/25 (52.0%)	LOQ–12.7	
3Ac-DON	maize	6/6 (100%)	63–643	[117]
		1/3 (33%)	10	[25]
		0/10 (0%)	-	[119]
		33–57%	0.3–368	[121]
	maize silage for feed and ready feed	71/1113 (6.4%)	LOD ***–527	[122]
	maize products	22–73%	0.3–105	[121]
	barley	14/34 (41.2%)	LOQ–23.6	[123]
	wheat	14/30 (46.7%)	LOQ–71.0	[123]
		1/6 (16.7%)	17	[117]
		3/5 (80.0%)	LOQ–50	[25]
	oat	24/31 (77.4%)	LOQ–2720	[123]
		6/6 (100%)	34–116	[117]
	bread	6/6 (100%)	29–51	[117]
	corn flakes	5/6 (83.3%)	29–52	[117]
	wheat flour	40/359 (11.1%)	0.6–3.6	[130]
	feed for swine	1/16 (6.3%)	14	[119]
	feed for poultry	2/15 (13.3%)	19–21	
	light beer	0/217 (0%)	-	[132]
	wheat beer	0/46 (0%)	-	
	dark beer	0/47 (0%)	-	
	bock beer	0/20 (0%)	-	
	alcohol free beer	0/19 (0%)	-	
	shandy beer	0/25 (0%)	-	
15Ac-DON	maize	6/6 (100%)	61–792	[117]
		0/3 (0%)	-	[25]
		6/10 (60%)	15–79	[119]
		48–90%	0.3–1734	[121]
	maize silage for feed and ready feed	128/1113 (11.5%)	LOD–2177	[122]
	maize products	52–92%	0.3–1519	[121]
	wheat	0/6 (0%)	-	[117]
		1/5 (20.0%)	20	[25]
	oat	2/6 (33.3%)	<LOQ–27	[117]
	bread	1/6 (16.7%)	18	
	corn flakes	1/6 (16.7%)	17	
	wheat flour	51/359 (14.2%)	2.0–11.1	[130]
	feed for swine	10/16 (62.5%)	10–34	[119]
	feed for poultry	7/15 (46.7%)	17–45	
3Ac-DON & 15Ac-DON	maize	-	<LOQ–11,900	[120]
	barley	1/24 (4.2%)	62	[124]
	wheat	34/99 (34.3%)	25–98	
	durum wheat	41/84 (49%)	LOQ–190	[128]
	triticale	20/20 (100%)	36–374	[124]
DON-3Glc	maize	6/6 (100%)	36–1003	[117]
		2/3 (67%)	<LOQ–70	[25]
		10/10 (100%)	<35	[118]
		8/10 (80%)	14–121	[119]
		-	<LOQ–1100	[120]
		33–83%	3–978	[121]
	maize silage for feed and ready feed	701/1113 (63.0%)	1.0–3159	[122]
	maize products	54–86%	3–844	[121]
	barley	25/34 (73.5%)	<LOQ–1300	[123]
		0/15 (0%)	-	[118]
		7/24 (29%)	43–277	[124]
	wheat	25/30 (83.3%)	<LOQ–922	[123]
		1/6 (16.7%)	18	[117]
		5/5 (100%)	50–200	[25]
		5/6 (83.3%)	43–737	[118]
		27/99 (27.3%)	40–356	[124]
		25/92 (27%)	16–138	[125]
		4/13 (31%)	<LOQ	[126]
		15/20 (75%)	100–1230	[127]
		5/5 (100%)	170–1040 **	[28]
	durum wheat	80/84 (94%)	LOQ–850	[128]
	triticale	1/5 (20.0%)	109	[118]
		15/20 (75.0%)	40–434	[125]
DON-3Glc	rye	0/12 (0%)	-	[118]
	oat	27/31 (87.1%)	<LOQ–6600	[123]
		6/6 (100%)	28–97	[117]
		5/11 (45.5%)	162–287	[118]
		0/4 (0%)	-	[124]
	bread	5/6 (83.3%)	26–29	[117]
	corn flakes	3/6 (50.0%)	24–28	
	white flour products	14/17 (82.4%)	5–30	[129]
	mixed flour and whole-corn products	28/36 (77.8%)	7–41	[129]
	breakfast cereals	6/7 (85.7%)	19–66	[129]
	snacks	28/34 (82.4%)	11–94	
	flours	15/22 (68.2%)	5–72	
	wheat flour	5/5 (100%)	110–770 **	[28]
		120/359 (33.4%)	0.2–15.7	[130]
	feed for swine	15/16 (93.8%)	6–80	[119]
	feed for poultry	14/15 (93.3%)	30–107	
	beer	19/20 (95%)	LOQ–27.5	[131]
	light beer	142/217 (65.4%)	<LOQ–81.3	[132]
	wheat beer	32/46 (69.6%)	<LOQ–28.4	
	dark beer	28/47 (59.6%)	<LOQ–26.2	
	bock beer	20/20 (100%)	2.4–33.3	
	alcohol free beer	9/19 (47.4%)	<LOQ–6.6	
	shandy beer	20/25 (80.0%)	<LOQ–7.9	
NIV	barley	25/34 (73.5%)	<LOQ–302	[123]
	wheat	13/30 (43.3%)	<LOQ–74.0	
	oat	22/31 (71.0%)	<LOQ–4940	
	wheat flour	4/4 (100%)	LOQ–140.6	[126]
NIV-3Glc	barley	21/34 (61.8%)	<LOQ–65.3	[123]
	wheat	13/30 (43.3%)	<LOQ–33.6	
	oats	6/31 (16.1%)	<LOQ–58.3	
HT-2	barley	12/34 (35.3%)	<LOQ–39.5	[123]
		8/8 (100%)	23–233	[42]
		18/18 (100%)	3–213	[133]
	wheat	4/4 (100%)	19–96	[42]
		19/30 (63.3%)	<LOQ–39.5	[123]
		7/9 (77.8%)	26–85	[43]
HT-2	oat	8/8 (100%)	11–187	[42]
		23/31 (74.2%)	<LOQ–1830	[123]
		8/9 (88.9%)	21–851	[43]
HT-2-Glc	barley	18/34 (52.9%)	<LOQ–38.5	[123]
		6/8 (75%)	- ****	[43]
		17/18 (94.4%)	0.6–162.8	[133]
	wheat	3/4 (75%)	- ****	[42]
		16/30 (53.3%)	<LOQ–15.9	[123]
	oat	6/8 (75%)	- ****	[42]
		5/31 (16.1%)	<LOQ–300	[123]
HT-2-di-Glc	barley	2/8 (25%)	- ****	[42]
	wheat	0/4 (0%)	- ****	
	oats	0/8 (0%)	- ****	
T-2	barley	8/8 (100%)	41–160	[42]
		18/18 (100%)	1–154	[133]
	wheat	4/4 (100%)	17–76	[42]
		5/9 (55.6%)	11–23	[43]
	oat	8/8 (100%)	32–165	[42]
		8/9 (88.9%)	10–377	[43]
T-2-Glc	barley	7/8 (87.5%)	- ****	[42]
		9/18 (50.0%)	0.1–14.5	[133]
	wheat	3/4 (75%)	- ****	[42]
	oat	6/8 (75%)	- ****	[42]
T-2-di-Glc	barley	0/8 (0%)	- ****	[42]
	wheat	0/4 (0%)	- ****	
	oat	0/8 (0%)	- ****	
T-2 & HT-2	maize	-	<LOQ–103	[120]
ZEN	maize	5/6 (83%)	59–1071	[117]
		-	<LOQ–15,700	[120]
	maize silage for feed and ready feed	884/1113 (79.4%)	20–11,192	[122]
	barley	2/34 (5.9%)	<LOQ–17	[123]
		16/24 (67%)	2–31	[124]
	wheat	14/30 (46.7%)	<LOQ–234	[123]
		5/6 (83.3%)	12–109	[117]
ZEN	wheat	47/99 (47.5%)	1–100	[124]
	triticale	20/20 (100%)	4–86	[124]
	oat	13/31 (41.9%)	<LOQ–675	[112]
		4/6 (66.7%)	13–85	[117]
		4/4 (100%)	5–15	[124]
	bread	5/6 (83.3%)	19–53	[117]
	corn flakes	5/6 (83.3%)	34–90	
ZEN-14Glc	maize	1/6 (17%)	274	[117]
	barley	6/34 (17.6%)	<LOQ–9.6	[123]
	wheat	2/30 (6.7%)	<LOQ–0.6	[123]
		0/6 (0%)	-	[117]
	oats	1/31 (3.2%)	<LOQ	[123]
		0/6 (0%)	-	[117]
	bread	2/6 (33.3%)	20–20	[117]
	corn flakes	0/6 (0%)	-	[117]
ZEN-16Glc	barley	8/34 (23.5%)	<LOQ	[123]
	wheat	2/30 (6.7%)	<LOQ–2.8	
	oat	18/31 (58.1%)	<LOQ–7.9	
ZEN-14S	maize	1/6 (17%)	51	[117]
	maize silage for feed and ready feed	471/1113 (42.3%)	2–4318	[122]
	barley	3/34 (8.8%)	<LOQ–26.1	[123]
	wheat	2/6 (33.3%)	11 (average)	[117]
		12/30 (40.0%)	<LOQ–22.5	[123]
	oats	1/6 (16.7%)	12	[117]
		9/31 (29.0%)	<LOQ–220	[123]
	bread	1/6 (16.7%)	24	[117]
	corn flakes	0/6 (0%)	-	[117]
α-ZEL	maize	6/6 (100%)	22–262	[117]
	barley	1/34 (2.9%)	<LOQ–0.6	[123]
		0/24 (0%)	-	[124]
	wheat	4/30 (13.3%)	<LOQ–0.7	[123]
		2/6 (33.3%)	16	[117]
		1/99 (1.0%)	8	[124]
	triticale	0/20 (0%)	-	[124]
α-ZEL-14Glc	oat	3/31 (9.7%)	<LOQ–2.2	[123]
		2/6 (33.3%)	<LOQ–68	[117]
		0/4 (100%)	-	[124]
	bread	2/6 (33.3%)	18–110	[117]
	corn flakes	2/6 (33.3%)	26–34	
	maize	1/6 (17%)	283	[117]
	barley	8/34 (23.5%)	<LOQ–5.1	[123]
	wheat	5/30 (16.7%)	<LOQ–4.4	[123]
		0/6 (0%)	-	[117]
	oat	0/6 (0%)	-	[117]
		0/31 (0%)	-	[123]
	bread	0/6 (0%)	-	[117]
	corn flakes	0/6 (0%)	-	
β-ZEL	maize	4/6 (67%)	12–103	[117]
	barley	1/34 (2.9%)	<LOQ–2.0	[123]
	wheat	2/30 (6.7%)	<LOQ–10	[123]
		1/6 (16.7%)	49	[117]
		30/99 (30.3%)	2–24	[124]
	triticale	0/20 (0%)	-	[124]
	oat	10/31 (32.3%)	<LOQ–6.0	[123]
		1/6 (16.7%)	46	[117]
		2/4 (50.0%)	6–9	[124]
	bread	3/6 (50.0%)	55–96	[117]
	corn flakes	4/6 (50.0%)	44–63	
β-ZEL-14Glc	maize	3/6 (50%)	92–193	[117]
	barley	1/34 (2.9%)	<LOQ–0.7	[123]
	wheat	0/6 (0%)	-	[117]
		0/30 (0%)	-	[123]
	oat	1/6 (16.7%)	20	[117]
		0/31 (0%)	-	[123]
	bread	0/6 (0%)	-	[117]
	corn flakes	0/6 (0%)	-	
α-ZEL & β-ZEL	maize	-	<LOQ–7970	[120]
ZEN-14Glc ZEN-14S α-ZEL-14Glc β-ZEL-14Glc	maize	-	<LOQ–9750 (total)	[120]

* Limit of quantification; ** Five wheat samples obtained by mixing of infected grain with mycotoxin-free grain in different proportions; *** Limit of detection, **** Relative content/relative peak are.
